# Formation of Twin-Free Single Phase β-In_2_Se_3_ Layers via Selenium Diffusion into InP(111)B Substrate

**DOI:** 10.1021/acs.cgd.4c00705

**Published:** 2024-11-04

**Authors:** Kaushini S. Wickramasinghe, Candice R. Forrester, Martha R. McCartney, David J. Smith, Maria C. Tamargo

**Affiliations:** † Department of Chemistry and Biochemistry, The City College of New York, New York, New York 10031, United States; ‡ Chemistry Program, CUNY Graduate Center, New York, New York 10016, United States; § Department of Physics, Arizona State University, Tempe, Arizona 85287, United States

## Abstract

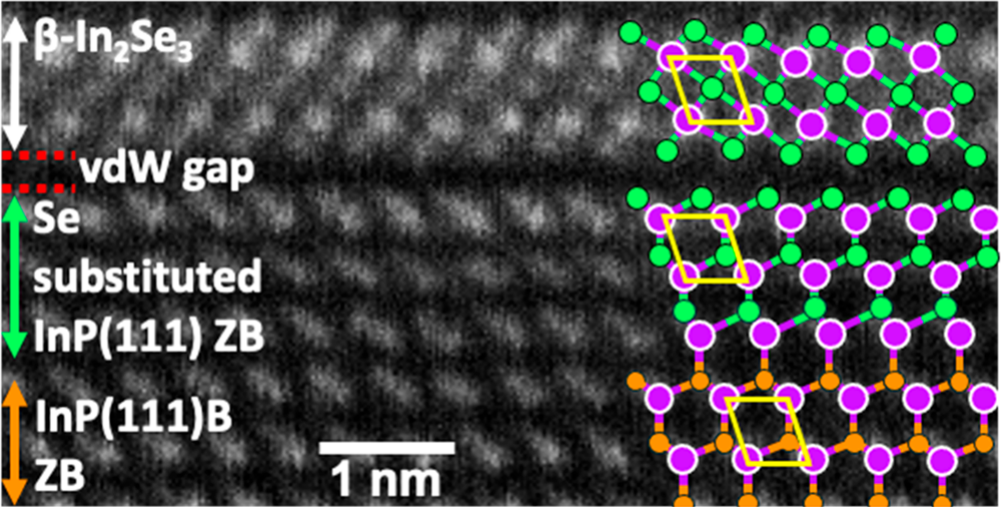

Indium selenide, In_2_Se_3_, has recently attracted growing interest due to its remarkable properties, including room temperature ferroelectricity, outstanding photoresponsivity, and exotic in-plane ferroelectricity, which open up new regimes for next generation electronics. In_2_Se_3_ also provides the important advantage of tuning the electrical properties of ultrathin layers with an external electrical and magnetic field, making it a potential platform to study novel two-dimensional physics. Yet, In_2_Se_3_ has many different polymorphs, and it has been challenging to synthesize a single phase material, especially using scalable growth methods, as needed for technological applications. We recently reported the growth of twin-free ultrathin layers of In_2_Se_3_ prepared by a diffusion driven molecular beam epitaxy approach, and twin-free Bi_2_Se_3_ layers grown on these unique virtual substrates. In this paper, we use aberration-corrected scanning transmission electron microscopy to characterize the microstructure of these materials. We emphasize features of the In_2_Se_3_ layer and In_2_Se_3_/InP interface which provide evidence for understanding the growth mechanism that leads to the twin-free and single phase In_2_Se_3_. We also show that this In_2_Se_3_ layer provides an ideal substrate for growth of twin-free Bi_2_Se_3_ with a nearly defect-free interface. This approach for growing high-quality twin-free single phase two-dimensional crystals using InP substrates is likely to be applicable to other technologically important materials.

## Abstract 2

Cross-sectional scanning transmission electron microscopy image showing atomic resolution of single phase β-In_2_Se_3_ layer grown via selenium (Se) passivation of InP(111)B substrate. Atomic models of β-In_2_Se_3_ and InP(111)B are overlaid with the real lattice depicting that indium atoms remain at the same positions in both lattices as shown by yellow box with the same dimensions. This also shows the result of selenium diffusing into InP and substituting for phosphorus (P) atoms while preserving the zinc-blende InP lattice right below the In_2_Se_3_/InP interface. These features provided evidence to uncover the growth mechanism which led to the formation of ultrathin layers of untwinned single phase β-In_2_Se_3_.

Two-dimensional (2D) van der Waals (vdW) materials have garnered much interest over the past decade because of the large variety of 2D compounds having potential applications for next generation electronic and optoelectronic devices.^1,2^ The class of 2D vdW materials began to evolve two decades ago, with the discovery of the semimetal graphene.^3,4^ Since then, the list of 2D materials has been rapidly expanding, and includes insulators such as hexagonal boron nitride (h-BN), transition metal dichalcogenides (TMDCs), such as MoS_2_, materials from group III–VI family (e.g., In_2_Se_3_, GaSe), and the black phosphorus family.^5^ Unlike 3D materials, it is easy to engineer the band gap using quantum confinement simply by changing the number of layers in the 2D material.^6^ Either by quantum confinement or by making heterostructures, many interesting physics topics can be studied, for example, superconductivity, magnetism, and charge density waves. Furthermore, the properties of the 2D material can be changed by applying an external electrical and magnetic field so that the material can be tuned into a different phase. For example, experiments showed that TiSe_2_, a semimetal, can be tuned into a superconducting phase by applying an external electric field.^7^

Indium selenide, In_*x*_Se_*y*_ has recently attracted renewed interest not only due to applications in thermoelectric devices to harness green energy^8^ but also owing to some of its remarkable properties such as room temperature ferroelectricity in the α-In_2_Se_3_ phase,^9^ outstanding photoresponsivity in β-In_2_Se_3_^10^, and exotic in-plane ferroelectricity in β’-In_2_Se_3_.^11,12^ Thus, In_2_Se_3_ has potential applications in energy harvesting, such as solar cells and photodetectors,^10^ as well as in electronic applications, such as ferroelectric semiconductor field effect transistors^9^ and phase change materials for data storage.^13^ In_2_Se_3_ also has been used as a buffer layer to improve the quality of Bi_2_Se_3_ grown by molecular beam epitaxy on sapphire.^14^ However, In_2_Se_3_ is a complex material with many different polymorphs, known as α, β, β′,γ, δ, and κ.^14−16^ Hence, it has been challenging to synthesize single phase In_2_Se_3_. There are a few reports of In_2_Se_3_ growth using physical vapor transport (PVT)^15^ which limits the crystal size, while chemical vapor deposition (CVD)^17^ and metalorganic chemical vapor deposition (MOCVD)^18^ are employed for scalable synthesis. However, improved crystal quality and single phase nature of the state-of-the-art material would be highly desirable. Among all scalable crystal growth methods, molecular beam epitaxy (MBE) offers distinct advantages since it can provide high crystallinity and controllability of thickness down to the few Ångstrom level. However, there are only a few reports of In_2_Se_3_ grown using MBE.^19−21^ Thus, epitaxial growth of scalable In_2_Se_3_ is still in its early stages. Hence, it is important to develop a scalable growth method that provides high quality and single phase In_2_Se_3_, especially on substrates that are currently in use for technological applications such as InP, GaAs, and Si.

Recently, our group reported the growth of twin-free In_2_Se_3_ on InP(111))B with abrupt and flat interfaces using a diffusion-driven MBE approach.^22^ Furthermore, this In_2_Se_3_ layer was then used as a substrate for the growth of highly crystalline twin-free Bi_2_Se_3_ and Sb_2_Te_3_ layers. Twin domains in these materials are highly deleterious for tera-hertz applications.^23^ Using our MBE approach, we grew ultrathin In_2_Se_3_ layers using a Se effusion cell but without an In effusion cell, making use of In from the substrate itself to form In_2_Se_3_. In our initial report we presented clear evidence of the different stages of the In_2_Se_3_ formation using *in situ* Reflection High Energy Electron Diffraction (RHEED) and presented evidence for the twin-free nature of In_2_Se_3_ grown on smooth nonvicinal InP(111)B substrates using high resolution X-ray diffraction (HR-XRD).^22^ The In_2_Se_3_ layer was then used as a template to achieve twin-free Bi_2_Se_3_, which was also investigated by HRXRD techniques.

In this study, we have used aberration-corrected scanning transmission electron microscopy (STEM) to conduct an in-depth structural analysis of the twin-free, single phase β-In_2_Se_3_ as-grown on smooth nonvicinal InP(111)B substrate, using the nonconventional MBE growth method developed in our laboratory,^22^ as well as the Bi_2_Se_3_ over layers. Details of the STEM measurements and specimen preparation can be found in the Supporting Information. We emphasize the microstructure of the In_2_Se_3_ layer and its interfaces, which provide the evidence that is needed to understand the growth mechanism of the twin-free In_2_Se_3_ layer grown by the diffusion driven MBE technique.^22^ Based on this analysis, we also propose the possible growth mechanism that resulted in the untwinned single phase material. Understanding and controlling the mechanism leading to these results should enable application of the same approach to the growth of other heteroepitaxial structures involving layered vdW materials on 3D crystalline substrates. It may also enable the possibility of stabilizing the In_2_Se_3_ α-phase which is of important technological interest owing to its ferroelectric properties but difficult to obtain.

A cross-sectional high-angle annular dark-field (HAADF) STEM image of the sample, which consists of two distinct layers, Bi_2_Se_3_ and In_2_Se_3_, with thicknesses of 65 and 7 nm, respectively, is shown in Figure 1a. The Bi_2_Se_3_ layer displays a defect-free nature without antiphase domains and no indication of twin boundaries or dislocations. Further cross-sectional HAADF-STEM images taken at widely separated locations on the same sample, confirm the excellent crystal quality (see Supporting Information Figure S1). By contrast, reports of twin-free Bi_2_Se_3_ grown on rough nonvicinal InP(111)B substrates have previously shown the presence of antiphase domains due to variations in substrate height^24^ and Bi_2_Se_3_ grown on flat InP(111)B has shown twin boundaries as well as dislocations.^25^Figure 1b shows well-ordered Bi_2_Se_3_ quintuple layers with an overlay of an atomistic model (Figure 1c), which confirms the presence of the rhombohedral crystal structure with the space group *R*3̅*m*. Additional HAADF-STEM images focusing on the In_2_Se_3_ layer of the sample are shown in Figures 1d and 2a, again confirming a well-ordered highly crystalline material which is free of structural defects.

**Figure 1:**
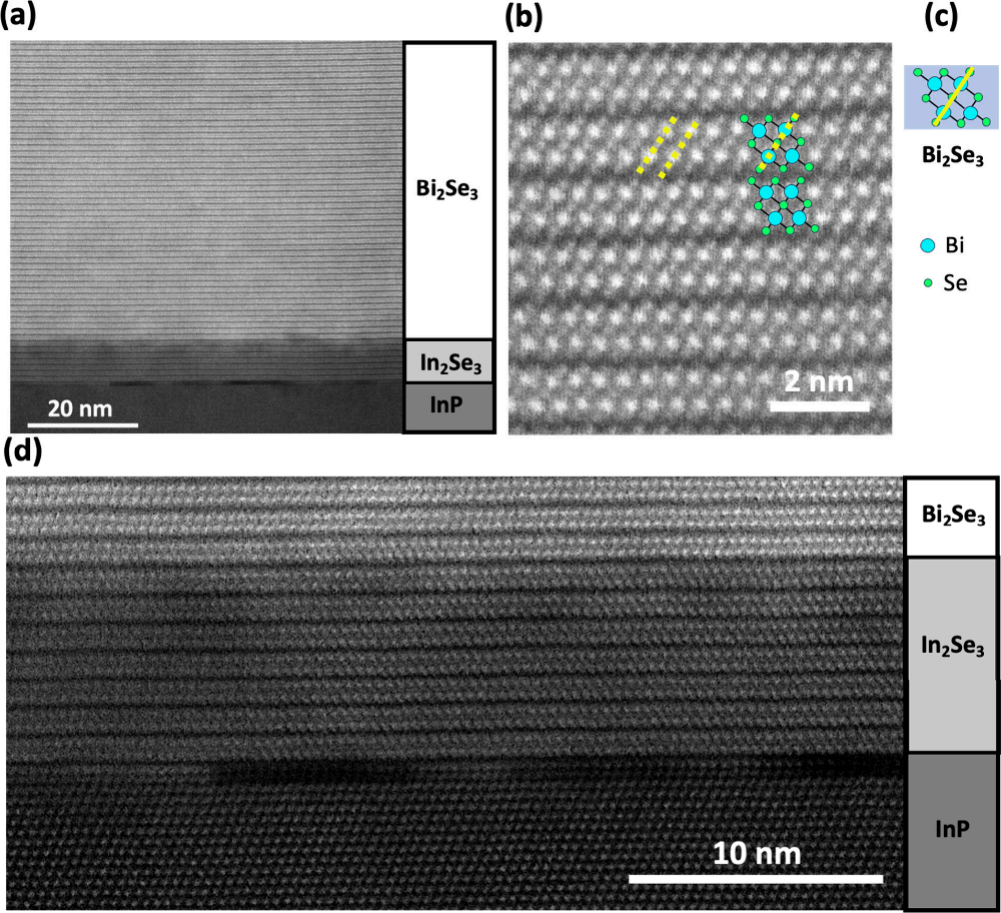
Cross-sectional HAADF-STEM images of Bi_2_Se_3_ (65 nm thick) grown on In_2_Se_3_/InP(111)B. (a) Highly ordered Bi_2_Se_3_ layer having an abrupt interface with the In_2_Se_3_ layer; (b) Enlarged image of Bi_2_Se_3_ layer with overlay of the atomistic model of Bi_2_Se_3_ quintuple layer. (c) Atomic model of B_2_Se_3_ quintuple layer consisting of 5 atoms. (d) Cross-sectional HAADF-STEM image showing well-ordered In_2_Se_3_ layer and interfaces with Bi_2_Se_3_ and InP.

**Figure 2:**
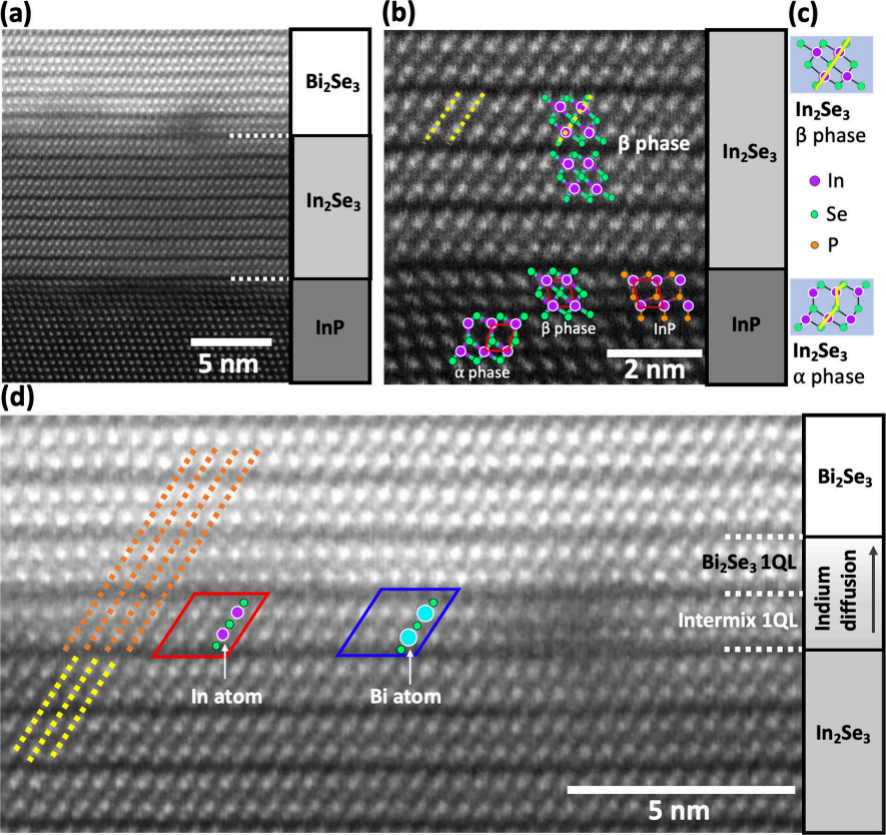
(a) Cross-sectional HAADF-STEM image of the sample taken at higher magnification than the image in Figure 1(d) showing the high crystallinity of β phase In_2_Se_3_ layer. (b) Enlarged image showing the atomic resolution of the β phase In_2_Se_3_ layer. An overlay of indium atoms in α and β phases of In_2_Se_3_ quintuple layer with indium atoms in InP lattice are also shown. Atomic model of InP(111)B lattice is shown next to the β-In_2_Se_3_ atomic model. (c) Atomic model of α and β phases of In_2_Se_3_ quintuple layer which consists of 5 atoms. (d) Enlarged image of Figure 2(a) showing In diffusion into Bi_2_Se_3_ layer at Bi_2_Se_3_/In_2_Se_3_ interface. The quintuple layer highlighted in the red box shows 5-atom chain In_2_Se_3_ whereas 5-atom chain Bi_2_Se_3_ is shown in the blue box. Orange and yellow lines are for guiding the eyes.

Previously, we found by using HR-XRD^22^ that these In_2_Se_3_ layers had a rhombohedral crystal structure with the space group R3̅m. It is well-known that In_2_Se_3_ with this space group has two principal polymorphs, namely the α and β phases.^15,16,26^ Since the lattice parameters of α-In_2_Se_3_ and β-In_2_Se_3_ are very similar, it is not possible using HR-XRD to identify the phase of the In_2_Se_3_ i.e., whether it is α, β, or a mixture of the two. Determining the In_2_Se_3_ phase by the commonly used Raman spectroscopy, or by photoluminescence spectroscopy, is also not feasible because the In_2_Se_3_ layer is too thin and the background signal from the InP wafer completely masks the signal from the layer. Thus, direct probing of the material structure, for example using STEM analysis, is essential.

Using cross-sectional HAADF-STEM images, as shown in Figure 2a, we established that the In_2_Se_3_ layer consists of a single phase. Furthermore, by using atomic-resolution HAADF-STEM images, such as shown in Figure 2b, and by overlaying atomistic models of α-In_2_Se_3_ and β-In_2_Se_3_ (see Figure 2c), we can conclude that the In_2_Se_3_ layer grown here is the β phase. The different phases of In_2_Se_3_ which can be formed readily, make it very difficult to achieve single phase material.^17^ However, these results indicate that the In_2_Se_3_ layers grown using our newly developed technique^22^ is capable of forming single phase β-In_2_Se_3._

Figure 2d shows an enlarged image of Figure 2a highlighting the interface between the In_2_Se_3_ and Bi_2_Se_3_ layers. In the image, we see evidence for indium diffusion into the Bi_2_Se_3_ layer as well as the formation of a single crystalline quintuple layer (1QL) with a mixture of both In_2_Se_3_ and Bi_2_Se_3_. In this intermixed layer, the 5-atom quintuple chain highlighted inside the red box is similar in size and shade to the In_2_Se_3_ region whereas those in the blue box have larger atoms at the In atomic column positions, which indicates these are Bi atoms that form Bi_2_Se_3_ quintuple chains in some regions. This intermixed layer is limited to one QL and the Bi_2_Se_3_ layer above then makes an abrupt interface with this layer. We do not observe any interfacial layer of poor crystalline quality, neither diatomic steps nor Bi_2_Se_4_ clusters as seen at the interface that forms when Bi_2_Se_3_ is grown on flat InP or on rough nonvicinal InP substrates, as reported elsewhere.^24,25^

The interface between the In_2_Se_3_ layer and the InP substrate has several notable features. As visible in the HAADF-STEM image shown in Figure 1a and the higher magnification image, Figure 1d, the interface has dark regions alongside the clear and sharp interface, suggesting the presence of some possibly defective regions. A significant contrast feature visible in some higher magnification cross-sectional bright field (BF) STEM images (Figures 3a and b) of the sample suggests significant Se diffusion into the substrate beyond the In_2_Se_3_/InP interface. The Se diffusion into the substrate was also evident in Energy Dispersive Xray Spectroscopy (EDS) analysis (see Supporting Information Figure S2). The presence of excess Se, as evident from the STEM contrast difference near the interface, does not however alter the crystal structure of the InP substrate, which remains zinc blende. This observation suggests that the In atoms remain fixed in their lattice sites during the In_2_Se_3_ formation process while Se diffuses into the substrate, displacing P. Then, with the special annealing step during the growth process, the Se atoms rearrange to form In_2_Se_3_ quintuple layers. Figure 3c is a schematic illustrating the In_2_Se_3_ formation steps during oxide desorption from the InP(111)B substrate surface in the unique MBE-based growth approach used here.^22^ By contrast, if we used conventional MBE to grow In_2_Se_3_, in which In and Se effusion cells would be used simultaneously, then In atoms would be mobile and able to form different phases as well as twin domains due to their closely similar thermodynamic properties.

**Figure 3:**
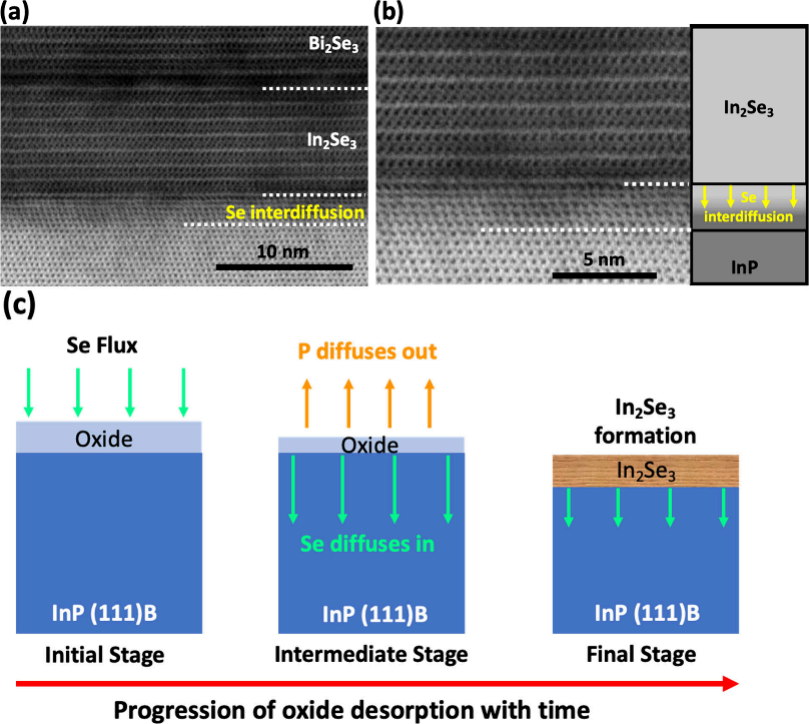
(a) Cross-sectional BF-STEM images highlighting diffusion of Se atoms into InP substrate during the In_2_Se_3_ growth process. (b) Cross-sectional BF-STEM image at higher magnification showing that the presence of excess Se atoms does not alter the crystal structure of the InP, which remains zinc blende. (c) Simple schematic showing Se atoms diffuse into InP substrate while P atoms diffuse out during the oxide desorption process and the formation of In_2_Se_3_ as the oxide desorption process is completed.

If this proposed mechanism, in which In atoms remain “anchored” through the process, is valid, then the preference to form the single β-In_2_Se_3_ phase over the α-In_2_Se_3_ phase can be understood. In Figure 2b, we have overlaid the In atoms in the α and β phases of In_2_Se_3_ quintuple layers on top of the In atoms in the InP(111)B lattice. We observe that the 4 In atoms in the red oblique square of the β-In_2_Se_3_ are well superimposed on top of the In atoms in the InP lattice whereas only the top two In atoms out of the four In atoms are superimposed in the red oblique square of the α-In_2_Se_3_, while the bottom two are shifted away from the In atoms in the InP lattice. This careful observation also leads to the conclusion that the In atoms remain anchored during the In_2_Se_3_ formation process. Based on these observations of cross-section STEM images as discussed above. The proposed growth mechanism for the formation of the twin-free and single phase β-In_2_Se_3_ is illustrated using a schematic atomic model in Figure 4, which first shows substitution of Se in the place of P in the zinc blende structure (Figure 4b) and then transformation of the Se-substituted 3D lattice into 2D β-In_2_Se_3_ (Figure 4c).

**Figure 4:**
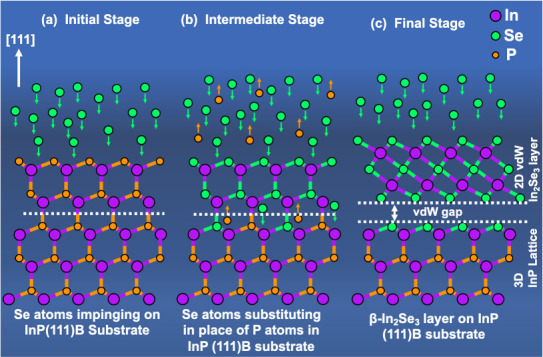
Proposed growth mechanism demonstrated using a cartoon atomic model for the formation of untwinned single phase In_2_Se_3_ layer based on the STEM image analysis. Orange arrows and green arrows represent desorption of P atoms from the substrate, and diffusion of Se atoms into the substrate, respectively. (a) The initial stage where the Se atoms are impinging on the substrate while the substrate is heated. (b) An intermediate stage where the Se atoms are diffusing into the substrate and substituting in place of P atoms while it remained zinc-blende. (c) The final stage where the Se substituted zinc-blende lattice transform into twin-free β-In_2_Se_3_ layers.

In summary, we have shown that high quality In_2_Se_3_ and Bi_2_Se_3_ crystalline layers that are fully twin-free and largely free of defects, can be achieved using InP(111)B substrates. STEM observations also show that the sample consists primarily of single phase β-In_2_Se_3_. Close observation of the InP/In_2_Se_3_ interface provides evidence for a mechanism in which Se first displaces P in the zinc blende InP, followed by a crystal structure transformation to the rhombohedral In_2_Se_3_ structure. This result implies that In atoms are not mobile during the transformation, thus resulting in twin-free In_2_Se_3_. This unique growth mechanism for In_2_Se_3_ also results in pure β-phase In_2_Se_3_. The resulting InP(111) substrate terminated by a thin In_2_Se_3_ layer results in an ideal surface (virtual substrate) for deposition of Bi_2_Se_3_ and other van der Waals materials. Understanding and controlling the mechanism of the In_2_Se_3_ formation would enable application of this approach to other heteroepitaxial structures involving layered, vdW materials on 3D crystalline substrates. It may also enable the possibility of stabilizing the different phases of In_2_Se_3_ which are of important technological interest owing to their exotic properties.

## Data Availability

All data are available from the corresponding author upon reasonable request.

## Supporting Information Available

Details of the materials and instrumentation used, details of the synthesis of the sample, an additional high-resolution scanning transmission electron microscopy (STEM) image taken at a different location of the sample, and energy dispersive X-ray spectroscopy (EDS) data of the sample cross section (PDF)
