# Supplementary material for: Formation of Twin-Free Single Phase β-In2Se3 Layers via Selenium Diffusion into InP(111)B Substrate
Source: Cryst Growth Des. 2024 Nov 4;24(22):9313–7. doi: 10.1021/acs.cgd.4c00705 (PMC11583199; doi:10.1021/acs.cgd.4c00705)
Supplement: Supplementary file 1 — cg4c00705_si_001.pdf [file cg4c00705_si_001.pdf]

Supporting Information

Formation of twin-free single phase β-In_2Se_3 layers via selenium diffusion into InP(111)B

Kaushini S. Wickramasinghe[a], Candice R. Forrester[a,b], Martha R. McCartney[c],

*David J. Smith[c], and Maria C. Tamargo[a,b]**

[a]Department of Chemistry and Biochemistry, The City College of New York, NY 10031, United States

[b]Chemistry Program, CUNY Graduate Center, New York, NY 10016, United States

[c]Department of Physics, Arizona State University, Tempe, AZ 85287, United States

*Email: mtamargo@ccny.cuny.edu, kskwick@gmail.com

1. Materials and Instrumentation

1.1. Molecular beam epitaxy

Samples were grown on smooth, non-vicinal Fe-doped InP(111)B ± 0.5° substrates with a phosphorus-terminated surface. A Riber 2300P molecular beam epitaxy (MBE) system with a base pressure of 5×10^{11} Torr was used to grow the material. The system was equipped with *in situ* reflection high-energy electron diffraction (RHEED) to facilitate monitoring of material growth in real time. High-purity (99.9999%) bismuth (Bi) and selenium (Se) were used as source materials. The Bi and Se fluxes were provided by a RIBER dual-zone effusion cell and a RIBER valved cracker cell for corrosive materiel (VCOR), respectively. An ultra-high vacuum (UHV) nude ion gauge positioned to intercept the path of the fluxes was used to measure beam equivalent pressures (BEP).

1.2. Scanning transmission electron microscopy

The samples were characterized using high-angle annular-dark-field (HAADF) and bright-field (BF) scanning transmission electron microscopy (STEM) imaging using a probe-corrected JEOL ARM200F operated at 200kV. Energy-Dispersive X-ray Spectroscopy was done with the same instrument using an Oxford Ultim Max EDS detector with 0.7 srad solid angle. Cross-section samples suitable for TEM observation were prepared using a dual-beam Thermo Fisher Helios 5G UX gallium ion milling system, initially at 30keV with subsequent thinning at 5keV and 2keV to reduce the amount of surface damage.

2. Synthesis

Using a diffusion driven MBE approach, we grew ultra-thin In_2Se_3 layers using a Se effusion cell without the use of an In effusion cell, making use of In from the substrate itself to form In_2Se_3. This layer was formed at a substrate temperature of T_{sub} = 505 °C with an Se overpressure of $1x10^{-5}$ Torr during the oxide desorption process without an indium (In) source cell. In our previous report[1] we presented clear evidence of the different stages of the In_2Se_3 formation using in situ Reflection High Energy Electron Diffraction (RHEED) and presented evidence for the twin-free nature of In_2Se_3 grown on smooth non-vicinal InP(111)B substrates using high resolution X-ray diffraction (HR-XRD) measurements[1].

A Bi_2Se_3 layer was then grown on the In_2Se_3 layer. The Bi_2Se_3 growth temperature (T_{sub}), growth rate and BEP ratio of Se to Bi were T_{sub} = 270 °C, ~0.5 nm/minute and ~100:1 respectively. A 1x1 RHEED pattern was observed after Bi_2Se_3 growth with increased intensity relative to the In_2Se_3 layer. These results were previously discussed in detail by Wickramasinghe et al[1]. The substrate temperature was measured using a thermocouple mounted behind the substrate holder. The sample analyzed here consisted of a 65-nm-thick Bi_2Se_3 layer on top of a ~ 7-nm-thick In_2Se_3 layer, both of which are twin-free[1].

3. HAADF images

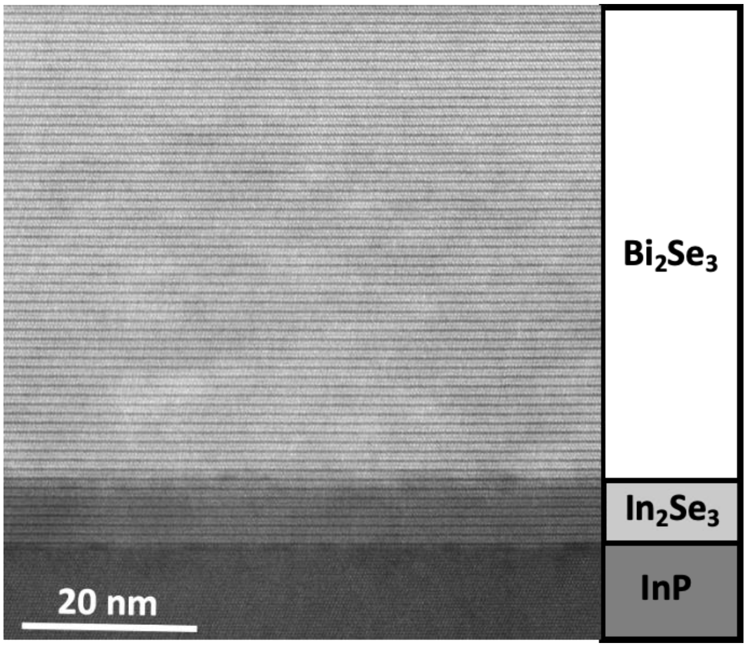

Figure S1. Cross-sectional HAADF-STEM images of Bi_2Se_3 (65-nm thick) grown on In_2Se_3/InP(111)B taken from a region several microns away from the area shown in Figure 1.a.

4. Energy Dispersive Xray Spectroscopy image

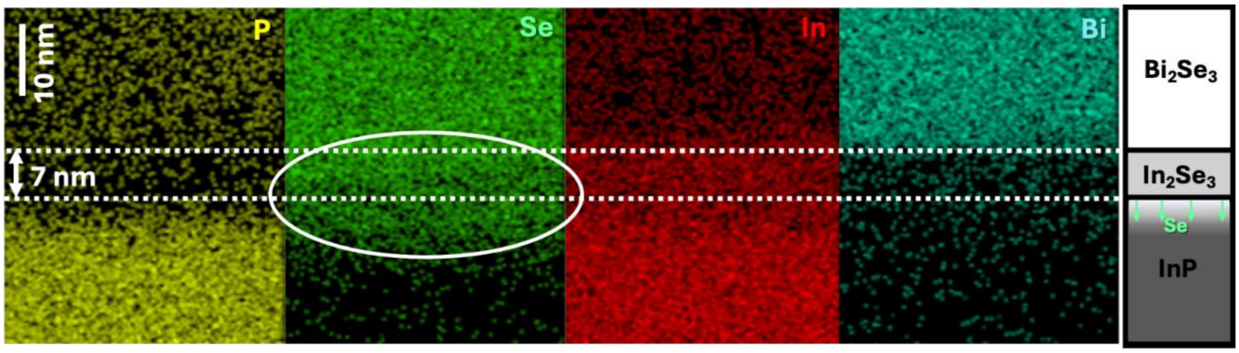

Figure S2. Energy Dispersive Xray Spectroscopy (EDS) montage showing the cross-sectional elemental distribution of P, Se, Bi and In. An abrupt Bi distribution is visible at the In_2Se_3/Bi_2Se_3 interface, defining the top of the In_2Se_3 layer, whereas a diffuse interface is visible for the P and Se distribution, confirming Se diffusion into the InP substrate at the In_2Se_3/InP interface, as highlighted with the white oval.

References

1. Wickramasinghe, K. S., Forrester, C. & Tamargo, M. C. Molecular Beam Epitaxy of Twin-Free Bi_2Se_3 and Sb_2Te_3 on In_2Se_3/InP(111)B Virtual Substrates. *Crystals* **13**, (2023).
